# Unmasking bias in the evidence ecosystem: a panoramic analysis of 311,751 meta-analyses using an artificial intelligence agent-based approach

**DOI:** 10.7189/jogh.16.03029

**Published:** 2026-09-04

**Authors:** Xi Chen, Zhenghang She, Shiqi Yang, Ming Chu, Yixin Zhou

**Affiliations:** 1 Department of Adult Joint Reconstructive Surgery, Beijing Jishuitan Hospital, Capital Medical University, Beijing, China; 2 Changping Laboratory, Beijing, China; 3 Department of Immunology, School of Basic Medical Sciences, Peking University, Beijing, China

**Keywords:** evidence synthesis, large language models, meta-analysis, research integrity, reporting bias, global health

## Abstract

Traditional secondary meta-analysis workflows are highly labour-intensive, time-consuming, and difficult to update in real time. Currently, there is a lack of comprehensive artificial intelligence frameworks capable of automating the entire meta-analysis workflow, including literature screening, data extraction, and quality assessment. Furthermore, a large-scale structured database for systematically analysing the global landscape of published meta-analyses remains unavailable. In this viewpoint, we aimed to evaluate the feasibility of large language models in automating meta-analysis workflows and develop the Meta-Analysis Screening, Transformation and Evaluation Review Agent (MASTER) agent; establish a large-scale Unified Meta-Analysis Repository (UMAR) and perform an exploratory panoramic analysis of the current evidence ecosystem; and develop an Agent-based Secondary Meta-analysis Platform (ASAP), integrating these capabilities. We subsequently applied the agent to process 311,751 meta-analysis records to establish the UMAR database. Building upon these resources, we developed the ASAP platform to support multimodal, automated meta-analysis workflows. In benchmark evaluations, the MASTER agent demonstrated high accuracy and stability in performing core automated meta-analysis tasks. The ASAP platform enabled automated literature retrieval, quality assessment, data extraction, and visualisation generation through predefined workflows. Here, we provide an initial exploration of the technical feasibility and scalability of artificial intelligence-driven automated meta-analysis.

## THE EVIDENCE PARADOX IN GLOBAL HEALTH

Systematic reviews and meta-analyses are the bedrock of evidence-based medicine (EBM) [1]. However, the field currently faces a dual crisis: a ‘velocity crisis’, where manual synthesis cannot keep pace with the rapid growth in publications over the last two decades, and a ‘transparency crisis’, where the sheer volume of meta-analyses makes large-scale methodological auditing nearly impossible [2].

Traditional natural language processing tools, while helpful, often struggle with the semantic complexity and nuanced reasoning required for high-level tasks like A Measurement Tool to Assess Systematic Reviews 2 (AMSTAR-2) quality assessment. The emergence of large language models (LLMs) may support tasks that extend beyond simple pattern matching, including agent-based evidence synthesis workflows [3–6].

## THE THEORY OF ARTIFICIAL INTELLIGENCE-DRIVEN SYNTHESIS: FROM TOOLS TO AGENTS

**Figure 1:**
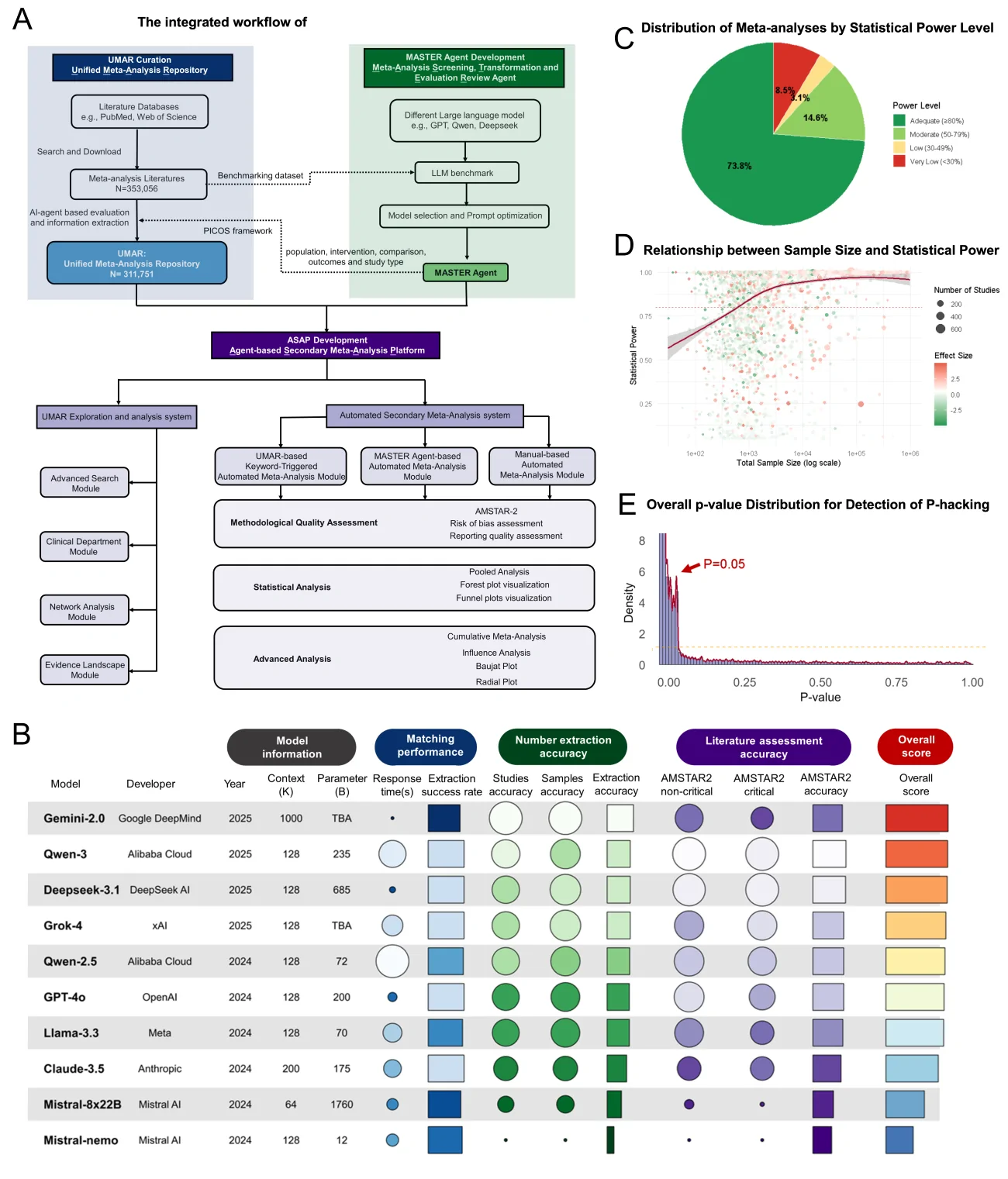
MASTER Agent framework and preliminary audit of the global meta-analysis ecosystem. **Panel A.** Integrated workflow of the MASTER Agent, UMAR repository construction, and ASAP platform, illustrating the transition from manual to artificial intelligence-assisted synthesis. **Panel B.** Benchmarking of 10 large language models across meta-analysis processing tasks, including Gemini-2.0 and GPT-4o. **Panel C.** Preliminary distribution of statistical power levels across the UMAR database. **Panel D.** Relationship between total sample size and statistical power, stratified by effect direction. Red indicates positive and green indicates negative effect directions. **Panel E.** Reported *P*-value distribution showing an apparent concentration near the 0.05 threshold, which may be consistent with selective reporting but may also reflect rounding conventions, model specifications, or extraction error. AI – artificial intelligence, AMSTAR-2 – A Measurement Tool to Assess Systematic Reviews 2, ASAP – Agent-based Secondary Meta-Analysis Platform, LLM – large language model, MASTER – Meta-Analysis Screening, Transformation and Evaluation Review Agent, UMAR – Unified Meta-Analysis Repository.

The core of our proposal is the transition from static software to autonomous ‘Agents’. Our theoretical framework, the Meta-Analysis Screening, Transformation and Evaluation Review Agent (MASTER Agent), integrates task planning, quality control, and self-correction into a unified workflow (**Figure 1**, Panel A).

Unlike traditional scripts, an LLM-based agent can use contextual cues to map disparate reporting styles to the population, intervention or exposure, comparator, outcomes, and study design framework within a prompted workflow. To compare candidate engines for this workflow, we conducted a systematic benchmark of 10 LLMs, including GPT-4o, Gemini-2.0, and Claude-3.5, to assess their accuracy and stability in processing meta-analytic data (**Figure 1**, Panel B). By utilising the top-performing models within a ‘Weighted Ensemble’ architecture, the MASTER Agent can theoretically mitigate ‘hallucination’ risks, creating a robust ‘digital auditor’ for global health data.

This viewpoint involves secondary analyses of a large publicly accessible evidence repository. We therefore followed JoGH’s GRABDROP and adhered to the five requirements (Table S1 in the **Online Supplementary Document**).

## MAPPING THE ECOSYSTEM: PRELIMINARY INSIGHTS FROM UNIFIED META-ANALYSIS REPOSITORY

To demonstrate the scalability of this framework, we constructed the Unified Meta-Analysis Repository (UMAR), a structured database of 311,751 meta-analyses processed *via* the MASTER Agent. While these results represent a preliminary assessment rather than a definitive census, they illustrate patterns in the evidence landscape that are difficult to examine manually at this scale.

Our pilot audit reveals potential systemic fragilities within the global evidence ecosystem. Preliminary metrics suggest that over one-quarter of the repository may consist of underpowered meta-analyses, with an apparent positive association between total sample size and achieved statistical power. Furthermore, an apparent concentration of reported *P*-values near the 0.05 threshold (**Figure 1**, Panels C–E) may be consistent with selective reporting, but may also reflect rounding conventions, model specifications, or extraction error. These findings suggest that artificial intelligence (AI)-assisted infrastructures may help identify macro-level methodological patterns that traditional peer review might overlook.

## ADDRESSING THE ‘BLACK BOX’: CHALLENGES AND VALIDATION

The transition to automated synthesis is fraught with methodological hurdles. We acknowledge that the reliability of automated extraction currently faces a ‘validation gap’. Scaling AI to millions of studies requires an expansive, ‘living’ benchmark that evolves alongside model capabilities.

A significant limitation remains the reliance on abstracts for large-scale parsing, which often omit granular data such as specific variance measures. Future iterations must prioritise full-text access through agentic Retrieval-Augmented Generation to ensure data integrity. Furthermore, AI-detected signals, such as the *P*-value distribution in **Figure 1**, Panel E, should be interpreted with caution; they may be consistent with selective reporting but may also reflect rounding conventions, model specifications, or extraction error. AI should thus be viewed as a ‘triage tool’ to flag high-risk research for human oversight.

## THE FUTURE: AGENT-BASED SECONDARY META-ANALYSIS PLATFORM AND REAL-TIME EBM

The realisation of real-time EBM depends on platforms like Agent-based Secondary Meta-Analysis Platform (ASAP). By integrating the UMAR database with the MASTER Agent’s reasoning capabilities, ASAP supports three modular workflows: fully automated keyword-triggered analysis, agent-assisted manual adjustment, and human-led refinement (**Figure 1****,** Panel A). This design supports continuous, reproducible evidence synthesis rather than a one-time ‘frozen’ event.

## VALIDATION GAP AND METHODOLOGICAL LIMITATIONS

The limitation of the MASTER is that we only apply the abstract in this agent. Many methodological elements required for power calculations, bias assessment, or AMSTAR-2 evaluation are not consistently available in abstracts. Reliance on abstracts may therefore introduce misclassification, especially for studies with incomplete reporting of effect sizes, variance estimates, inclusion criteria, and analytical models. For this reason, the current system should be viewed as a triage and prioritisation tool rather than a replacement for expert-led systematic review.

Future work should include full-text retrieval, agentic retrieval-augmented generation, expert-curated gold-standard data sets, formal benchmarking across language models, transparent ensemble weighting, and reproducible version-controlled pipelines. In particular, the ability of LLM agents to perform AMSTAR-2 assessment should be evaluated independently before such outputs are used for methodological judgement.

## GOVERNANCE AND ETHICAL CONSIDERATIONS

Large-scale automated evidence synthesis raises important ethical and governance concerns. These include propagation of biases from indexed literature, language and database coverage bias, errors introduced by automated extraction, limited transparency of model reasoning, and potential overreliance on AI-generated conclusions in clinical or policy settings. Any AI-assisted evidence platform should therefore maintain human oversight, transparent version control, audit trails, reproducible code, and clear accountability for errors. AI agents should support, rather than replace, expert interpretation.

## CONCLUSIONS

AI-assisted evidence synthesis may help address the growing mismatch between the scale of biomedical literature and the limited capacity for manual evidence auditing. The MASTER Agent, UMAR, and ASAP platform illustrate a possible direction for scalable, continuously updated evidence mapping. However, the findings presented here remain preliminary and hypothesis-generating. Before AI-driven systems can be used for routine evidence appraisal, they require rigorous validation against expert human assessment, full-text data extraction, transparent benchmarking, and robust governance mechanisms. At present, their most appropriate role is to assist human reviewers by flagging areas that may warrant closer methodological scrutiny.

## Additional material


Online Supplementary Document

## Data Availability

**Data availability:** ASAP is publicly available at https://chatgptmodel.shinyapps.io/UMAR/. Code is publicly available at https://github.com/chenxi199506/ASAP.
