# Supplementary material for: Unmasking bias in the evidence ecosystem: a panoramic analysis of 311,751 meta-analyses using an artificial intelligence agent-based approach
Source: J Glob Health. 2026 Sep 4;16:03029. doi: 10.7189/jogh.16.03029 (PMC13542800; doi:10.7189/jogh.16.03029)
Supplement: Supplementary file 1 [file jogh-16-03029-s001.pdf]

Supplement to: Chen X, She Z, Yang S, Chu M, Zhou Y. Unmasking bias in the evidence ecosystem: a panoramic analysis of 311,751 meta-analyses using anartificial intelligence agent-based approach. J Glob Health. 2026;16:03029.

Online Supplementary Document

Table S1. Adherence to JoGH's Guidelines for Reporting Analyses of Big Data Repositories Open to the Public (GRABDROP).

GRABDROP item	Purpose	Authors' response
1. Please list all papers published by each co-author in previous three years that were based on secondary analysis of a big data repository	Preventing publications from possible 'paper mills' and authorships granted without genuine contribution	None. None of the co-authors published papers during the three years preceding submission that were based on secondary analysis of a big-data repository.
2. Please explain the key elements of your study design and the use of the available datasets that make your study an original scientific contribution	Preventing duplicate publication where published information is reorganised and republished	The study makes an original contribution by integrating three linked components: systematic benchmarking of ten large language models for meta-analysis-related tasks; construction and audit of the Unified Meta-Analysis Repository (UMAR), containing 311,751 records meeting the automated inclusion criteria; and development of the MASTER Agent and the ASAP platform for automated screening, data extraction, quality assessment, statistical analysis, and visualisation. The combination of model benchmarking, repository-scale evidence mapping, and an operational automated meta-analysis workflow is the principal innovation.
3. Please list all publications that addressed similar research questions in the same dataset and indicate where you cited them in your paper	Preventing duplicate publication where other groups have already conducted similar research in the same dataset	None. To the best of the authors' knowledge, no previous publication has addressed similar research questions using the UMAR dataset.

GRABDROP item	Purpose	Authors' response
4. Please explain how you addressed multiple testing through an appropriately rigorous statistical threshold and indicate this in the methods section	Preventing publication of false positive associations which are of low value unless a rigorous statistical method accounted for multiple testing	Multiple-testing correction was applied. Statistical significance was assessed using corrected P values with a threshold of 0.05. The correction procedure and threshold are reported in the Methods.
5. Please declare to what extent have AI chatbots been used in developing your paper and to which parts of the paper did they contribute	Preventing the improper use of AI chatbots in developing the key elements of the paper and ensuring reproducibility and transparency	Generative AI was used only to assist with language polishing. It did not contribute to the study design, data collection, statistical analysis, interpretation of results, or generation of scientific conclusions. All authors reviewed and approved the final text and remain fully responsible for the accuracy and integrity of the manuscript.

SUPPLEMENTARY METHODS

Benchmark Dataset

We curated and organised publicly available secondary datasets as the gold standard for our benchmarking study. The dataset comprised 97 systematic reviews and meta-analyses. Data extraction was independently performed by two reviewers using standardised forms, with any discrepancies resolved through team discussion to ensure high accuracy. In addition, both reviewers conducted a rigorous risk-of-bias assessment for each article using AMSTAR-2, a validated tool for evaluating systematic reviews.

AMSTAR-2 comprises 16 items (7 critical items and 9 non-critical items), each rated as “Yes,” “Partial Yes,” or “No.” Critical domains include: protocol registration, adequacy of search strategy, justification for excluding individual studies, risk-of-bias assessment, appropriateness of meta-analytic methods, consideration of risk of bias in interpretation, and evaluation of publication bias. Reviews were categorised according to overall confidence as High (0 critical weaknesses and <3 non-critical weaknesses), Moderate (1 critical weakness and <3 non-critical weaknesses), Low (>1 critical weakness and <3

non-critical weaknesses), or Critically Low (>1 critical weakness and ≥3 non-critical weaknesses). This curated dataset provides a reliable benchmark for evaluating the objective performance of large language models (LLMs) in automated meta-analysis tasks.

Benchmarking Workflow

We selected 10 large language models (LLMs) available through the OpenRouter platform for comparative evaluation. These models included: GPT-4o (OpenAI), Qwen-2.5, Qwen-3 (Alibaba Cloud), Claude-3.5 (Anthropic), Mistral-8x22B, Mistral-Nemo (Mistral AI), Deepseek-3.1 (DeepSeek AI), Gemini-2.0 (Google DeepMind), Grok-4 (xAI), and Llama-3.3 (Meta). Key model specifications, including parameter count and context length, are summarised in Figure 1, of the main manuscript. The overall benchmarking workflow is depicted in Figure 1, of the main manuscript.

Each article in the benchmark dataset was individually input into every LLM, and outputs were aggregated and analysed (**Figure 1, Panel B, in the main manuscript**). Evaluation comprised three main dimensions: (1) Matching Performance: This included response time (seconds) and extraction success rate. Response time was defined as the average duration for each LLM to process an article and return results, reflecting model efficiency in automated pipelines. Extraction success rate measured whether the LLM returned non-empty outputs for requested data points or assessment conclusions. (2) Data Extraction Accuracy: We assessed the ability of each LLM to accurately extract critical numeric information from article text, focusing on sample size and number of included studies. Because the evaluation relied on abstracts rather than full texts, we also analysed the impact of incomplete information on model performance. Accuracy was calculated as the proportion of correctly extracted numeric values relative to the total number of values. (3) Literature Assessment Accuracy: We simulated human review by instructing each LLM to perform AMSTAR-2 evaluations for every article. Model outputs were compared against human-derived gold standards for all 16 AMSTAR-2 items, with separate consideration of critical and non-critical domains. All benchmarking scripts and code have been made publicly available on GitHub, ensuring reproducibility and transparency of the evaluation

process.

MASTER Agent Development

Based on the comprehensive benchmarking results, Gemini-2.0 was selected as the core reasoning engine for constructing the MASTER Agent (Meta-Analysis Screening, Transformation, and Evaluation Review Agent), a domain-specific tool designed for secondary meta-analysis. The development process aimed to transform a general-purpose large language model into a reliable and efficient professional instrument, capable of fully automated literature screening, data extraction, and quality assessment.

The first stage involved domain-specific prompt engineering and workflow design to guide the model toward meta-analysis tasks. Rather than simple question-answering, this process employed multi-step reasoning strategies. For example, in applying the AMSTAR-2 tool, the model was instructed to evaluate all 16 items sequentially, document supporting or opposing evidence for each item, and synthesise an overall confidence rating. This structured reasoning framework enhanced both the transparency and accuracy of quality assessments. To further improve task comprehension, a few-shot learning approach was incorporated. Carefully selected examples representing diverse study types, such as randomised controlled trial meta-analyses and observational study syntheses, as well as common challenges like incomplete abstracts, were embedded in the prompts. Exposure to these "standard answers" enabled the model to better understand the task requirements and expected output format. Moreover, all outputs were enforced to conform to a predefined structured format, such as JSON, ensuring compatibility with downstream modules and enabling fully automated integration within the broader platform workflow.

The second stage focused on sampling parameter optimisation and stability testing. To balance the model's creative flexibility, necessary for handling complex or unconventional studies, with the consistency required for automated data extraction and evaluation, extensive grid searches and iterative testing were conducted. Temperature

parameters ranging from 0.1 to 0.7 were explored, revealing that lower temperatures (0.2) minimised stochastic variation, critical for precise numeric extraction, whereas slightly higher temperatures (0.3) improved interpretive performance for nuanced AMSTAR-2 evaluations. The top-p parameter was set to 0.9, restricting the model to high-probability tokens and further enhancing output stability and professional terminology consistency. After over 500 iterative tests, the optimal parameter combination was identified, resulting in inter-class correlation coefficients exceeding 0.95 across task modules, indicating exceptionally high stability and reproducibility of outputs.

Finally, the optimised MASTER Agent was encapsulated as a modular, reusable service and integrated into the Agent-based Secondary Meta-Analysis Platform (ASAP). Within ASAP, the MASTER Agent serves as the core data processing and evaluation unit, operating within a comprehensive workflow where upstream modules perform literature retrieval and screening, and downstream modules conduct data synthesis and visualisation. Upon receiving a manuscript, the agent executes an automated pipeline: parsing the text, extracting key elements according to the population, intervention or exposure, comparator, outcomes, and study design (PICOS) framework, accurately capturing numerical data, performing AMSTAR-2 assessments, and returning standardised JSON results to the platform database. This design enables seamless end-to-end automation while maintaining accuracy and reproducibility, forming the foundation of ASAP's intelligent secondary meta-analysis capabilities.

Construction of the Unified Meta-Analysis Repository (UMAR)

We conducted a systematic retrieval of twelve major biomedical literature databases, including CINAHL, Cochrane, Embase, MEDLINE, Emcare, ProQuest Health and Medical Complete, ProQuest Nursing and Allied Health Source, PsycINFO, Scopus, Sport Discus, EBSCOhost, and Web of Science, covering all records from database inception through June 30, 2025. The search strategy used “meta-analysis” as the primary keyword, combined with title and abstract field restrictions and publication-type filters to maximise coverage of

relevant literature. Literature screening and data extraction were fully automated using the previously developed MASTER Agent. Initially, 353,056 records were retrieved across all databases. The MASTER Agent then performed automated quality assessment and relevance screening based on predefined algorithms that considered study design clarity, methodological completeness, and statistical correctness. Concurrently, natural language processing techniques identified and excluded irrelevant records, such as non-medical publications or misclassified methodological papers, and records failing the automated inclusion criteria.

For records that passed these screening steps, the MASTER Agent performed structured data extraction according to the PICOS framework, capturing population (P), interventions or exposures (I), comparators (C), outcomes (O), and study design (S). In addition, key metadata were automatically extracted, including the number of included studies, total sample size, effect estimates with confidence intervals, first author, publication year, associated clinical department, and disease focus. Following this process, a total of 311,751 records meeting the automated inclusion criteria were incorporated into the Unified Meta-Analysis Repository (UMAR), forming a comprehensive, computable knowledge base for subsequent analyses.

Statistical Analysis of the Unified Meta-Analysis Repository (UMAR)

To investigate the statistical characteristics associated with different effect directions, we conducted a series of multidimensional analyses. Statistical precision was defined as the reciprocal of the confidence interval width. Stratified analyses were performed to examine the influence of total sample size—categorised into four levels (<100, 100–1,000, 1,000–10,000, ≥10,000)—and the number of included studies—categorised into three groups (1–5, 6–20, >20)—on precision. Group comparisons were conducted using the Wilcoxon rank-sum test. To further assess the independent effects of effect direction, study count, and mean sample size on effect magnitude and precision, we fitted multivariable linear regression models controlling for clinical department as a fixed effect, with study

count square-root transformed to normalise its distribution.

Potential selective reporting was explored using four complementary indicators: uniformity of p-value distributions, cliff-edge effects near the 0.05 significance threshold, excessive significance detection, and a weighted scoring system combining these metrics. Statistical power of individual meta-analyses was systematically assessed, prioritising standard error calculations derived from reported effect sizes and confidence intervals, and employing p-value–based imputation when necessary. Conservative estimates under small-effect assumptions were additionally provided. All statistical analyses were performed in R (tidyverse for data processing, ggplot2 for visualisation), with fixed random seeds and rigorous quality control procedures to ensure reproducibility. Key findings were cross-validated using multiple methods, and statistical significance was uniformly defined at 0.05.

Visualisation of the Unified Meta-Analysis Repository (UMAR)

To facilitate exploration and interactive visualisation, all UMAR outputs were integrated into a **Shiny-based web platform** (https://chatgptmodel.shinyapps.io/UMAR/), providing users with multi-dimensional access to department- or disease-specific meta-analysis data. The platform comprises four core modules. First, the **UMAR Search module** enables dynamic keyword search and advanced filtering for precise data retrieval. The interface incorporates a responsive search panel supporting Boolean logic (AND/OR) with up to ten keywords, multi-dimensional filters covering disease type, clinical department, interventions, comparators, outcomes, study type, and participant characteristics, and a real-time statistical dashboard. Queries are processed server-side, first searching the metadata database globally and then applying filters to produce interactive tables.

Second, the **UMAR Department Analysis module** provides an interactive visualisation system for exploring meta-analysis characteristics across clinical departments. Users can select departments from a sidebar displaying summary statistics. Four analytical views are provided: a disease-distribution bar chart showing the ten most frequently analysed diseases per department, a bubble plot depicting sample size, study count, and proportion of significant results for the top fifteen diseases, a funnel plot using quantile-based denoising (excluding the outermost 1% of values) to assess publication bias, and an effect-size bubble plot relating effect estimates to statistical significance (-log10 p-value) with a 0.05 significance threshold indicated. All visualisations employ consistent aesthetic themes with gradient colour schemes and professional layout, and support dynamic updates based on department selection.

Third, the **UMAR Network Analysis module** constructs an interactive clinical department–disease association network using the igraph and visNetwork packages in R. Users may upload CSV data or utilise built-in samples. The system generates bipartite networks with nodes representing departments or diseases, and edges indicating association

strength. Users can filter networks by minimum edge weight, node degree, and maximum node count, and customise visualisation parameters including layout algorithms (e.g., Fruchterman-Reingold, Kamada-Kawai), node and edge colours, label display, and scale. Both interactive and static network views are provided, and the system computes network statistics including node and edge counts, density, average degree, diameter, clustering coefficient, degree distribution, and centrality rankings.

Finally, the **UMAR Evidence Map module** presents clinical evidence distribution through an interactive bubble-plot interface built with Shiny and Plotly. Interventions are mapped to the horizontal axis and diseases to the vertical axis, with bubble size representing total sample size and colour encoding outcome type. The visualisation supports zooming and panning, and clicking a bubble reveals study details—including abbreviations, PMID links, disease classification, interventions, comparators, outcomes, clinical department, effect estimates with 95% confidence intervals and p-values, study count, and sample size—alongside mini forest plots displaying effect estimates and confidence intervals. Data filtering is responsive, enabling dynamic updates according to clinical department or outcome type. All interface components adhere to a unified aesthetic design, featuring card-style layouts and coordinated colour schemes to ensure consistency and usability.

Development of the UMAR-Based Keyword-Triggered Automated Meta-Analysis Module

The platform was implemented with a modular architecture encompassing three core components: keyword search, visualisation, and statistical analysis. The user interface employs a responsive layout, with a multi-tiered search panel on the left-hand side. Users can construct complex search strategies using a dynamic keyword system, supporting both "OR" (match any keyword) and "AND" (match all keywords) Boolean operators. The search scope spans multiple dimensions, including participant characteristics, interventions,

comparators, outcomes, disease type, clinical department, literature identifiers, and outcome categories.

Visualisation features provide customisable display options, including forest plot text size (10–18 pt), point size adjustment (2–8 units), and control over study-level statistics and overall effect diamond plots. The platform incorporates an intelligent data validation mechanism that automatically identifies and flags records with complete effect size data. For each query, real-time metrics are calculated, including total matches, the number of records with complete effect size data, and their proportion. Interactive tables support sorting, filtering, and export in multiple formats.

The forest plot module automatically processes effect estimates and confidence intervals, dynamically scaling point sizes according to sample size, and provides an overall effect estimate. The funnel plot module integrates publication bias assessments, including contour visualisation and Egger test results. Meta-regression functionality allows users to select explanatory variables such as total sample size, publication year, or clinical department; linear models are automatically fitted and statistical summaries provided. The platform leverages reactive programming to ensure seamless synchronisation between user interactions and visualisation outputs. The keyword management system supports dynamic addition or removal of up to ten search terms. All visualisations are rendered with ggplot2 to ensure publication-quality output, and data and graphics can be exported as CSV and high-resolution PNG files (300 DPI), respectively.

MASTER Agent-based Automated Meta-Analysis Module

The platform is implemented with a modular architecture, enabling users to execute all critical steps of systematic review and meta-analysis through a unified interface. The workflow begins with data import, supporting CSV-formatted research data uploads, including study title, authors, publication year, study design, sample size, population,

interventions, and outcomes. Users are required to provide a DeepSeek API key and specify the meta-analysis topic, after which the platform automatically applies predefined thresholds for relevance (0–10 points) and quality (high/medium/low) to execute study selection.

The AI-assisted assessment process is guided by a hierarchical prompt engineering strategy, in which the model is framed as a domain expert in systematic review and meta-analysis. User-provided prompts include the meta-analysis topic, study data, and detailed evaluation requirements. Study screening involves assessing topic relevance, providing inclusion/exclusion recommendations with justification, and evaluating data completeness. Quality assessment is conducted using the AMSTAR-2 instrument, covering study design appropriateness, risk of bias, reporting completeness, and statistical methodology, culminating in an overall quality rating. Effect size extraction is fully automated, with the system calculating Hedges' g, odds ratios, risk ratios, or mean differences along with 95% confidence intervals.

To enhance efficiency, the platform supports parallel processing, allowing users to configure batch sizes from one to ten studies. A built-in JSON repair mechanism addresses common formatting issues in API outputs, including removal of Markdown code blocks, correction of unquoted string values, and numeric value adjustments. Real-time progress monitoring provides users with complete visibility and control over the analytical workflow. Advanced statistical analyses are performed using a random-effects meta-analysis framework, generating overall effect estimates, heterogeneity indices (I^2, Tau^2), and significance metrics. Visualisation tools include enhanced forest plots displaying individual study effect sizes, confidence intervals, and weights; funnel plots with contour lines to assess publication bias; cumulative meta-analysis plots illustrating trends as studies are sequentially included; influence plots to identify high-impact studies; Baujat plots for heterogeneity diagnostics; and radial plots as alternative representations of effect size and precision. The system automatically generates comprehensive summary reports detailing the study selection process, quality assessment distribution, effect size aggregation, and

heterogeneity evaluation. All results are exportable in multiple formats, including structured CSV files for data and high-resolution (300 DPI) PNG files for figures suitable for publication.

Manual-based Automated Meta-Analysis Module

The platform also accommodates manual data curation workflows, supporting uploads in Excel, CSV, or text formats (tab- or comma-delimited). An intelligent column detection algorithm automatically identifies key variables including effect sizes, confidence interval bounds, authorship, publication year, study name, and sample size, while allowing manual correction to ensure accurate column mapping. Uploaded data undergoes rigorous standardisation, with columns renamed to standardised fields (e.g., effect_size, ci_lower, ci_upper), calculation of standard errors ($SE = (CI_{upper} - CI_{lower}) / (2 \times 1.96)$), and generation of unified study labels by combining author and year information. A data validation module evaluates missing values, consistency of data types, and reasonable numeric ranges, providing detailed reports on completeness, missingness patterns, and overall data quality.

Meta-analyses can be executed using multiple modelling approaches, including random-effects (REML, default), fixed-effects, maximum likelihood, and DerSimonian-Laird methods. The platform automatically calculates pooled effect estimates, 95% confidence intervals, significance testing (z-tests), and heterogeneity indices (I^2, Tau^2, Q statistic). Publication bias is assessed via funnel plot asymmetry using the Egger test. Visualisation modules include professionally styled forest plots that highlight individual study effects and the overall pooled estimate, with customisable options for text size, point dimensions, and colour schemes. Funnel plots support contour lines and Egger test visualisation. Additional plots include cumulative meta-analysis charts, influence plots to identify studies with substantial impact on overall estimates, Baujat plots to detect sources

of heterogeneity, and radial plots offering alternative perspectives on effect size and precision. All results are exportable in multiple formats, with structured CSV files containing standardised variables and derived metrics, and high-resolution (300 DPI) PNG files for figures. Complete analysis reports integrate data overview, statistical results, heterogeneity diagnostics, and publication bias assessments, providing a fully publication-ready output.
